# Assessing the impact of transplant site on ovarian tissue transplantation: a single-arm meta-analysis

**DOI:** 10.1186/s12958-023-01167-6

**Published:** 2023-12-12

**Authors:** Baoli Xie, Jiaxu Li, Yingqin Huang, Fu Hang, Qianwen Hu, Jiaxin Yu, Aiping Qin

**Affiliations:** 1 Center of Reproductive Medicine, The First Affiliated Hospital of Guangxi Medical University, Nanning, 530021 China; 2 Center for Reproductive Medicine, Maternal and Child Health Hospital in Guangxi, Guangxi, 530021 China

**Keywords:** Ovarian tissue cryopreservation (OTC), Ovarian tissue transplantation (OTT), Transplant sites, Reproductive outcomes, Single-arm meta-analysis

## Abstract

**Background:**

Survival rates of young women undergoing cancer treatment have substantially improved, with a focus on post-treatment quality of life. Ovarian tissue transplantation (OTT) is a viable option to preserve fertility; however, there is no consensus on the optimal transplantation site. Most studies on OTT are nonrandomized controlled trials with limited sample sizes and uncontrolled statistical analyses, leaving the question of which transplant site yields the highest chance of achieving a live birth unanswered.

**Objective:**

This meta-analysis aimed to assess the effect of different ovarian transplant sites on postoperative reproductive outcomes.

**Methods:**

We adhered to the PRISMA Reporting Items for Systematic Reviews and Meta-Analyses recommendations. Systematic searches were conducted in PubMed, Embase, Web of Science, and the Cochrane Library from inception to September 17, 2023. The inclusion criteria were as follows: (1) women who underwent OTT with a desire for future childbirth, and (2) reports of specific transplant sites and corresponding pregnancy outcomes. The exclusion criteria included the inability to isolate or extract relevant outcome data, case reports, non-original or duplicate data, and articles not written in English.

**Results:**

Twelve studies (201 women) were included in the meta-analysis of cumulative live birth rates (CLBR) after OTT. The CLBR, which encompasses both spontaneous pregnancies and those achieved through assisted reproductive technology (ART) following OTT to the ovarian site, was 21% (95% CI: 6–40, I^2^: 52.81%, random effect). For transplantation to the pelvic site, the live birth rate was 30% (95% CI: 20–40, I^2^: 0.00%, fixed effect). Combining transplantation to both the pelvic and ovarian sites resulted in a live birth rate of 23% (95% CI: 11–36, I^2^: 0.00%, fixed effect). Notably, heterotopic OTT yielded a live birth rate of 3% (95% CI: 0–17, I^2^: 0.00%, fixed effect).

**Conclusion:**

Pregnancy outcomes were not significantly different after orthotopic ovarian transplantation, and pregnancy and live birth rates after orthotopic OTT were significantly higher than those after ectopic transplantation.

**Registration Number:**

INPLASY202390008.

**Supplementary Information:**

The online version contains supplementary material available at 10.1186/s12958-023-01167-6.

## Background

Globally, the number of young women diagnosed with cancer has increased. According to crude and age-standardized incidence rates per 100,000 women worldwide, 0.9 million new cases of cancer were diagnosed in 2020 [1]. The likelihood that these people will survive has increased because of improvements in cancer therapies, such as chemotherapy and radiation. Women aged 15–39 years and children under 14 years had 5-year survival rates of 86.7% and 84%, respectively [2]. Cancer survivors are interested in techniques that enhance their quality of life after treatment [3].

The American Society for Reproductive Medicine now supports the fertility preservation methods of mature oocyte cryopreservation after ovarian stimulation, embryo cryopreservation, and ovarian tissue cryopreservation (OTC) [4, 5]. In OTC, the cryopreserved tissue is kept in vials, thawed, and transplanted back to restore fertility and endocrine function after cancer treatment is completed, and the patient is declared disease-free by their oncologist. Ovarian tissue may be surgically transplanted into the surviving ovary (orthotopic), pelvic sidewall, subcutaneously or intramuscularly (heterotopic), or any combination of these locations. Because of this surgery, women can now have biological children while returning their ovarian hormone levels to normal. Numerous young girls and women have undergone OTC [6, 7]. The first successful autotransplantation of frozen-thawed ovarian cortical tissue occurred in 1999, whereas the first successful transplant of fresh human ovaries in monozygotic twins was reported in 2005 [8]. Since the first live birth following ovarian tissue transplantation (OTT) [9], the literature has reports of more than 200 live births [10].

Considerable evidence supports the viability of frozen and thawed ovarian tissues. However, the rate of OTT is substantially low, and the amount of clinical expertise is limited. A recent meta-analysis of three centers reported a 50% pregnancy rate [11]. The usual time for the ovarian tissue to regain normal endocrine function after transplantation is approximately 2–5 years, although a large majority of patients (95%) do so [12]. Functional longevity and the reserve of ovarian tissue are connected [13].

Different reproductive outcomes can occur after OTT. Some women become pregnant on their own [14]; however, in others, despite numerous in vitro fertilization (IVF) procedures, they are unable to conceive [15]. Importantly, adverse effects are probably underreported, and several studies found that women who underwent OTT and IVF had worse ovarian responses to external stimulation [16, 17]. According to a recent systematic evaluation of 20 trials, women undergoing OTT and IVF had overall pregnancy rates (PR) and live birth rates (LBR) of 14.4% and 10.7% per cycle, respectively [17]. Similar rates of spontaneous conception from the OTT to the ovarian site (52%, n = 47) and to a peritoneal pelvic location (50%, n = 6) have been described [11], and in another report, 31% and 35%, respectively [18]. Because of the limited experience from non-ovarian locations, it is challenging to evaluate their effectiveness in comparison with the ovarian site.

Thus, questions remain regarding the clinical effectiveness, best grafting site, tissue quantities, and other variables that may predict success. The exploration of these concerns is crucial because many women return for ovarian tissue grafting after the ovarian tissue is frozen. To date, there is no consensus on the optimal transplantation site. Most OTT studies were nonrandomized controlled trials with small sample sizes and uncontrolled statistical analyses. Consequently, it is unclear which transplant site has the best probability of producing live deliveries. This meta-analysis aimed to assess the effect of different ovarian transplant sites on postoperative reproductive outcomes.

## Methods

### Literature search strategy and eligibility criteria

The search methodology, criteria for selection, data extraction, criteria for evaluating the quality of the data, and statistical analyses mentioned below were all predefined in the version-controlled documents. The Preferred Reporting Items for Systematic Reviews and Meta-Analyses criteria served as the basis for the execution and reporting of this prospectively registered (INPLASY202390008). From the beginning of the study through to September 17, 2023, systematic searches were performed in PubMed, Embase, Web of Science, and the Cochrane Library. Using a mix of free terms, variations, and regulated vocabulary (such as MeSH terms/descriptors), a search strategy was built on the following themes: pregnancy outcome, reproductive outcome, pregnancy rate, delivery rate, reproductive outcome, pregnancy, ovarian transplantation, and ovarian tissue transplantation (Supplementary Data File S1).

The primary outcome was the LBR, which was calculated by dividing the number of births that resulted in at least one live infant by the initial number of females that had undergone an ovarian tissue graft to restore fertility. The secondary outcomes were the proportion of pregnant women and frequency of miscarriages.

Studies were considered for inclusion in this meta-analysis if they satisfied the following criteria: (1) women who underwent OTT with a desire for future childbirth, and (2) reports of specific transplant sites and corresponding pregnancy outcomes. The exclusion criteria included the inability to isolate or extract relevant outcome data, case reports, non-original or duplicate data, and articles not written in English.

### Selection process and data collection

Two reviewers examined all article titles and abstracts separately to identify which studies should be further evaluated. They also omitted any citations that they felt were irrelevant. The authors, institutions, publication titles, and study findings had no bearing on the initial screening. A discussion with a third reviewer helped to clarify any issues or points of contention. The reviewers discussed and agreed on any points of disagreement or doubts. Information was obtained from the included papers using a data extraction form created by the authors. The following information was gathered to describe the included studies: baseline characteristics of the females (number, age, indication for OTC); study characteristics (country, study type, inclusion and exclusion criteria, study duration); and baseline characteristics of the males (number, age, indication for OTC). Furthermore, we gathered the following information on the ovarian tissue: surgical methods, ovarian transplant site, number of ovarian transplants, number of autografts, duration of ovarian endocrine function, age at retrieval, age at transfer/transplantation, duration of storage, time from transfer/transplantation to pregnancy, duration of follow-up, maternal age at delivery, gestational age at delivery, and primary and secondary outcomes after spontaneous conception and after IVF.

### Assessment of study quality

Additionally, all included studies were evaluated and the necessary data were retrieved separately by two researchers. The quality of non-controlled trials was assessed using the Newcastle-Ottawa Scale (NOS) [19]. The three criteria used to assess the research were participant selection, comparability, and outcome ascertainment (Supplementary Data File S2).

### Data analysis

Data from the cohort studies were included in the meta-analysis only if the ovarian transplant site had been used in more than one female. Each result was analyzed independently. The meta-analysis includes all relevant studies with quantitative cumulative live birth rate (CLBR) data. A meta-analysis was performed only when two or more studies were included. No missing information has been replaced.

STATA 14.2 software (StataCorp LP, College Station, TX, United States) was used to evaluate all data in this meta-analysis. The I^2^ statistic and chi-square test were used to assess heterogeneity. Statistically significant differences are indicated by p < 0.1. A random-effects model was used when considerable heterogeneity (p-value < 0.1 and I^2^ > 50%) was observed. In addition, a fixed-effects model was used [20]. Additionally, sensitivity analysis was performed to assess the consistency and dependability of the combined data.

## Results

**Fig. 1:**
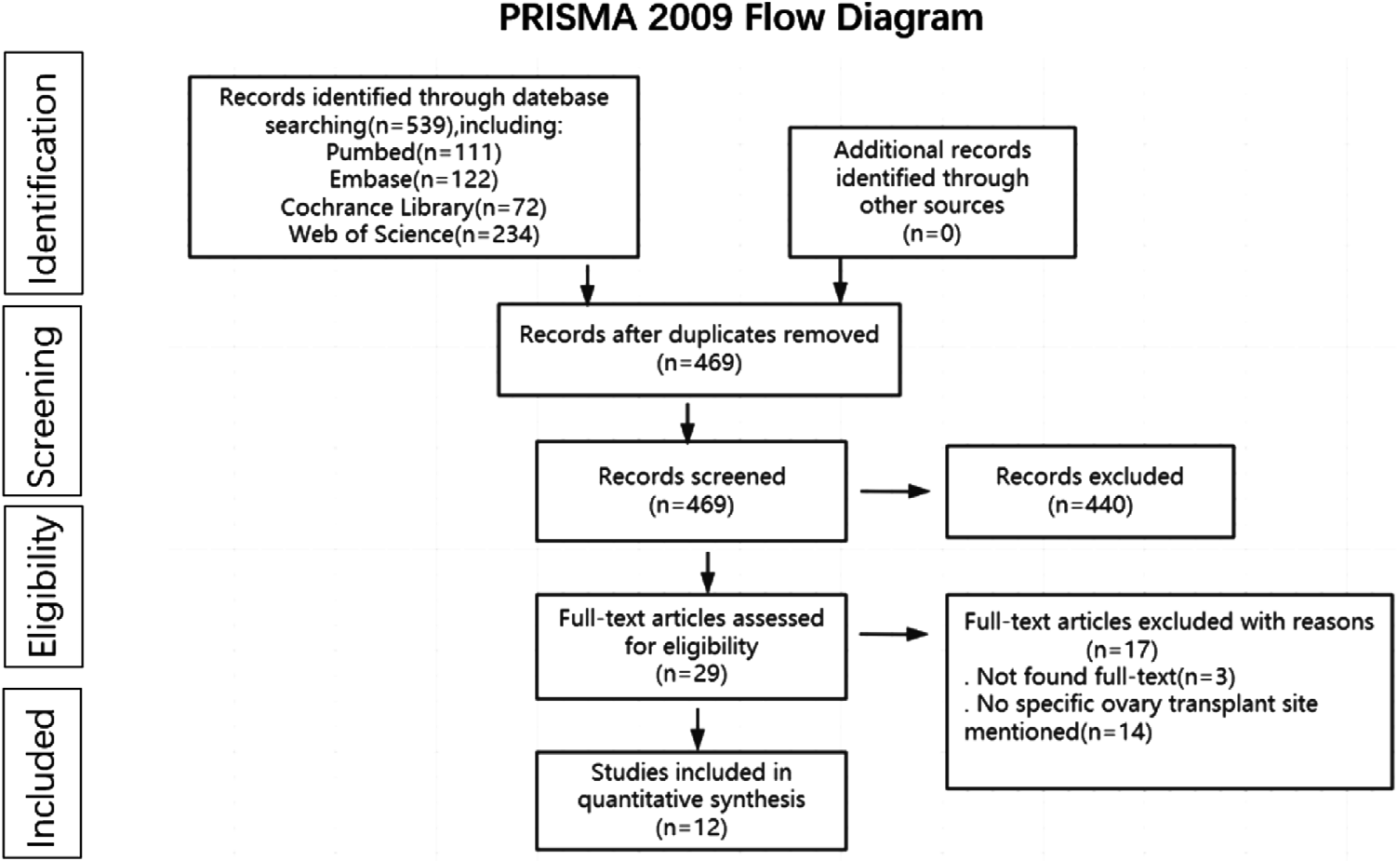
Study flowchart. Taken from Moher D, Liberati A, Tetzlaff J, Altman DG, The PRISMA Group (2009). Preferred reporting items for systematic reviews and metaAnalyses: The PRISMA statement. PLoS Med 6(7):e1000097. 10.1371/journal.pmed1000097. For more information, visit www.prisma-statement.org

Our search revealed 539 reports (PubMed = 111, Embase = 122, Web of Science = 234, and Cochrane Library = 72), of which 70 were duplicates; 29 reports were potentially eligible and the full text was retrieved after screening the titles and abstracts. Among the 29 articles searched in the full text, three were excluded because the full text could not be searched, 14 were excluded because there was no specific transplant site, and the remaining 12 studies involving 201 individuals (comprising 257 total ovarian transplants) were finally included in the meta-analysis. Figure 1 shows a flowchart of the selection process. Table 1 provides information on the characteristics of each included study.

**Table 1:** Characteristics of the studies included in the meta-analysis

Reference	Country	Study design	Pathology	Sample size	Number of OTT	Mean age at OTC (years)	Mean age at OTT (years, first time)	Transplantation sites	Quality score
Marine Leflon et al. 2022	France	Cohort study	Lymphoma:8; Breast cancer:1	9	9	26.2 ± 3.9	32.5 ± 3.88	O + P:8; P:1	6
R. Imbert et al. 2014	Belgium	Cohort study	HL:2; Breast cancer:1; Colorectal cancer:1; Sickle cell disease:1; NHD:1	6	7	25.1 ± 6.9	30.1 ± 5.8	O + P + SC:1; O + SC:4; O + P:2	6
C. Poirot et al. 2019	France	Cohort study	Borderline tumour:3; NHD:9; Cervix cancer:1; Pseudomyxomal peritonei:1; Shwachman–Diamond syndrome:1; Sickle cell disease:1; β Thalassemia:1; HL:13; Ewing sarcoma:1	31	38	26.2 ± 5.8	33.5 ± 4.8	O + P:34; HT:4	6
M. Vatel et al. 2021	France	Cohort study	HL:5; NHD:2; Sickle cell disease:1; Pseudo-myxoma:1; Invasive cervical carcinoma:1; Ovarian borderline tumor:1;	11	16	26.3 ± 6.0	32.6 ± 5.4	P:14; HT:2	6
Matthia W et al. 2017	Germany	Cohort study	hematologic neoplasia:17; breast carcinoma:10; germ cell or borderline ovarian tumor:4; anal cancer:3; premature ovarian failure:2; ovarian cancer:1; cervical cancer:1	38	39	NA	34.8 (range 27–44 years).	P:39	5
Ina Marie Dueholm Hjorth et al. 2020	Denmark	Cohort study	breast cance:14; hematologic malignancies:8; other malignancies:5; benign disease:1	28	36	29.8 ± 5.2	34.0 ± 5.1	P:18; O:10; P + O:8	6
Jana Liebenthron et al. 2019	Germany	Cohort study	Breast cancer:13; HL:8; NHD:5; Sarcoma:1; Gynecological cancer:1; Other types of malignancies:1; Lupus erythematosus:1	30	30	31.1 ± 5.0	34.8 ± 4.3	P:27; O + P:3	6
Ellen J. Hoekman et al. 2019	Netherlands	Cohort study	Breast cancer:3; HL:2; NHD:1; Ewing’s sarcoma:1	7	9	27.0 ± 4.7	33.4 ± 5.8	O:8; O + P:1	6
Debra Gook et al. 2021	Australia	Cohort study	due to a cancer diagnosis:14	17	25	27.4	33.4	A:9; O:9: P:7	6
Tryde Schmidt et al. 2011	Denmark	Cohort study	NHD:2; Hodgkin:4; Ewing’s sarcoma:1; Breast cancer:1; paroxysmal nocturnal hemoglobinuri:1; Aplastic anemia:1; Cervical cancer:1; hemolytic urinary syndrome:1	12	17	NA	28.4 ± 4.9	O:8; O + A:5; O + A + P:2; O + P:1; P:1	6
Tine Greve et al. 2012	Demark	Cohort study	NHD:2; HL:4; Ewing sarcoma:1; Breast cancer:3; PNH:1; Aplastic anemia:1	12	19	29.3 ± 5.3	31.5 ± 5.4	O:11; O + A:5; O + A + P:2; O + P:1	6
Genia Rozen et al. 2021	Australia	Cohort study	oncological (81%); medical (19%) indications	11	12	27.3 ± 6.6	34.7 ± 5.8	A:2; P:5; O:2; A + O:1; O + P:1; A + P:1	6

O, ovarian site; P, pelvic wall; A, anterior abdominal wall; SC, subcutaneous site; HT, heterotopic transplantation; OTC, ovarian tissue cryopreservation; OTT, ovarian tissue transplantation.

PNH, paroxysmal nocturnal hemoglobinuria; HL, Hodgkin lymphoma; NHD, non-Hodgkin disease.

Twelve OTT studies were included, all of which were retrospective cohort studies [21–32]. Of those who underwent OTT, 88% had malignant tumors, 12% had other medical indications, and 22.4% underwent a second transplant. The mean age at the time of ovarian freezing was 28.25 ± 5.7 years, and the evaluation age at the time of the first ovarian transplant was 33.52 ± 5.0 years. Five studies reported orthotopic transplantation and seven reported orthotopic and heterotopic transplantation. Of the 12 studies included in our analysis, two them [22, 25] exclusively featured patients who achieved spontaneous pregnancies, while in five studies [26, 29–32], patients received postoperative assisted reproductive technology (ART) treatment only. In the remaining five studies [21, 23, 24, 27, 28], patients experienced both spontaneous pregnancies and ART procedures after surgery.

### Primary outcome

#### CLBR after OTT

**Fig. 2:**
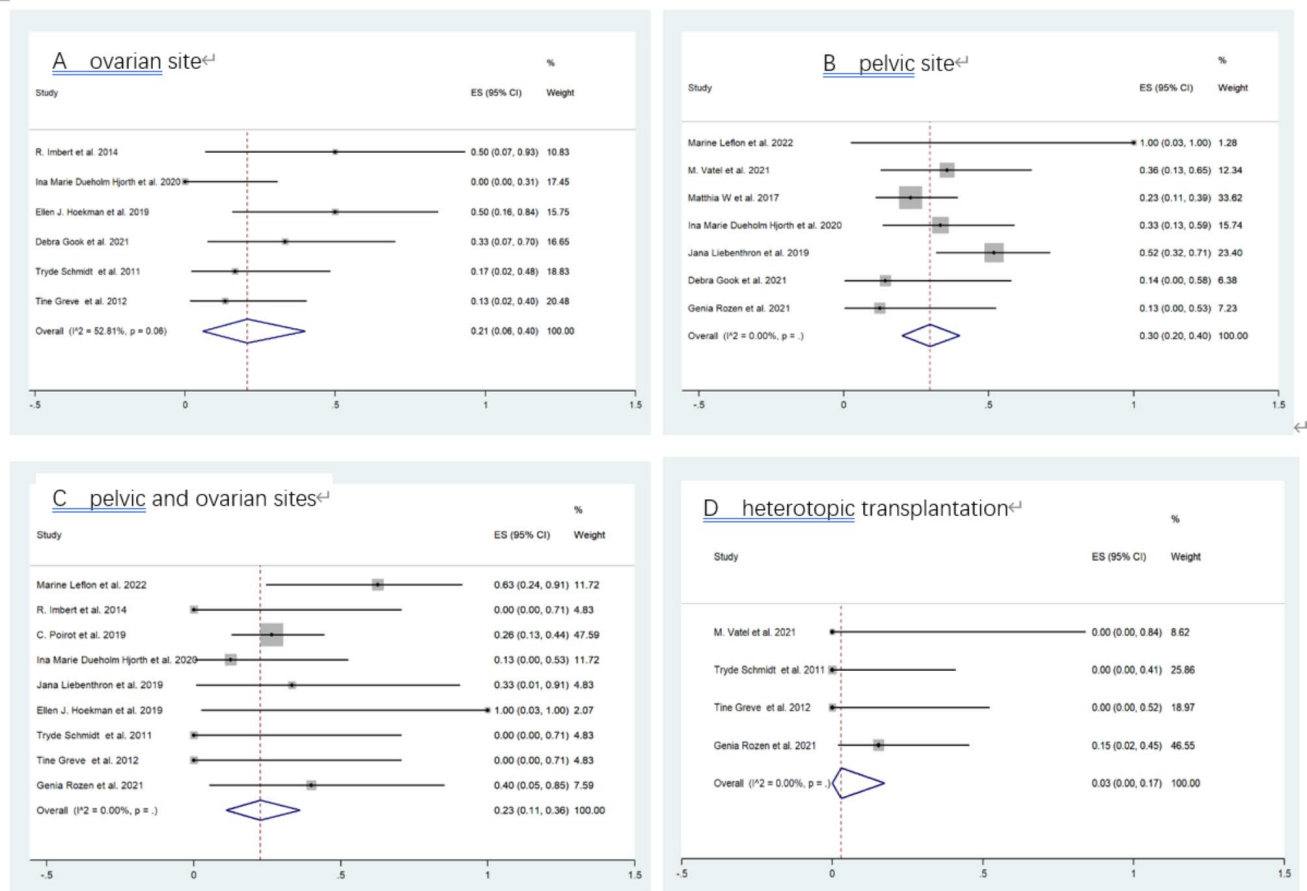
Cumulative live birth rate (CLBR) following ovarian tissue transplantation (OTT) to different sites. Analysis of CLBR following transplantation to the (**A**) ovarian site, (**B**) pelvic site, (**C**) pelvic and ovarian sites, and (**D**) heterotopic transplantation

Twelve studies (201 women) were included in the meta-analysis of CLBR after OTT. The CLBR, which encompassed both spontaneous pregnancies and those achieved through ART following OTT to the ovarian site, was 21% (95% CI: 6–40, I^2^: 52.81%, random effect). For transplantation to the pelvic site, the live birth rate was 30% (95% CI: 20–40, I^2^: 0.00%, fixed effect). Combining transplantation to both the pelvic and ovarian sites resulted in a live birth rate of 23% (95% CI: 11–36, I^2^: 0.00%, fixed effect). Notably, heterotopic OTT yielded a live birth rate of 3% (95% CI: 0–17, I^2^: 0.00%, fixed effect) (Fig. 2).

#### Spontaneous LBR after OTT

Seven studies were included in the meta-analysis of spontaneous LBR after OTT. For transplantation to the ovarian site, the spontaneous LBR was 16% (95% CI: 3–35, I^2^: 0.00%, fixed effect), whereas transplantation to the pelvic site yielded a LBR of 31% (95% CI: 21–42, I^2^: 0.00%, fixed effect). When transplantation occurred at both the pelvic and ovarian sites, the spontaneous LBR was 26% (95% CI: 12–41, I^2^: 0.00%, fixed effect). Importantly, no spontaneous pregnancies were observed after ectopic ovarian transplantation.

#### LBR after OTT and IVF

Ten studies involving OTT followed by ART were included in the analysis. Following ovarian transplantation and subsequent ART, the LBR was 10% (95% CI: 2–22, I^2^: 0.00%; fixed effect). For transplantation to the pelvic site followed by ART, the LBR was 18% (95% CI: 7–31, I^2^: 0.00%, fixed effect). When transplantation occurred at both the ovarian and pelvic sites, followed by ART, the LBR was 5% (95% CI: 0–27, I^2^: 0.00%, fixed effect). In cases of ectopic transplantation followed by ART, the LBR was 3% (95% CI: 0–17, I^2^: 0.00%; fixed effect).

### Secondary outcome

#### Percentage of women with at least one pregnancy after IVF and spontaneous pregnancy

Following transplantation to the ovarian site, 32% of the women (95% CI: 20–44, I^2^: 0.00%, fixed effect) achieved at least one pregnancy. Transplantation to the pelvic site resulted in 33% (95% CI: 17–52, I^2^: 59.74%, random effect), whereas transplantation to both the pelvic and ovarian sites yielded 23% (95% CI: 10–39, I^2^: 0.00%, fixed effect). Heterotopic OTT showed a percentage of 5% (95% CI: 0–16, I^2^: 0.00%, fixed effect).

#### Percentage of women with at least one spontaneous pregnancy

Specifically examining spontaneous pregnancies, transplantation to the ovarian site led to 26% of the women (95% CI: 9–46, I^2^: 0.00%, fixed effect) achieving at least one spontaneous pregnancy. For transplantation to the pelvic site and to both the pelvic and ovarian sites, the percentages were 32% (95% CI: 14–54, I^2^: 70.92%, random effect) and 38% (95% CI: 10–71, I^2^: 60.46%, random effect), respectively. Notably, no spontaneous pregnancies were observed after heterotopic OTT.

#### Percentage of women with at least one pregnancy after IVF

Among women who underwent OTT followed by IVF, the percentage of women who achieved at least one pregnancy varied. Following transplantation to the ovarian site, 9% of women (95% CI: 0–25, I^2^: 0.00%, fixed effect) achieved pregnancy. Transplantation to the pelvic site yielded 28% (95% CI: 7–54, I^2^: 65.63%, random effect), whereas transplantation to both the pelvic and ovarian sites yielded 15% (95% CI: 0–40, I^2^: 0.00%, fixed effect). Heterotopic ovarian tissue transplantation yielded 7% (95% CI: 0–24, I^2^:0.00%, fixed effect).

### Miscarriage

#### The percentage of women with miscarriage after IVF and spontaneous pregnancy

After transplantation to the ovarian site, the miscarriage rate was 10% (95% CI: 2–21, I^2^: 0.00%, fixed effect). The rate with transplantation to the pelvic site was 11% (95% CI: 0–27, I^2^: 62.34%, random effect). Combined transplantation to both the pelvic and ovarian sites resulted in a rate of 8% (95% CI: 1–18, I^2^: 0.00%, fixed effect). Heterotopic ovarian tissue transplantation showed a miscarriage rate of 7% (95% CI: 0–23, I^2^: 0.00%; fixed effect).

#### The percentage of women with miscarriage following spontaneous pregnancy

The miscarriage rate after transplantation to the ovarian site was 5% (95% CI: 0–19, I^2^: 0.00%, fixed effect). The rate with transplantation to the pelvic site was 12% (95% CI: 0–33, I^2^:7 7.90%, random effect). Transplantation to both the pelvic and ovarian sites yielded a rate of 3% (95% CI: 0-112, I^2^: 0.00%, fixed effect).

#### Percentage of women with miscarriage after IVF

After transplantation to the ovarian site, the miscarriage rate was 0% (95% CI: 0–9, I^2^: 0.00%, fixed effect). The rate with transplantation to the pelvic site was 14% (95% CI: 2–30, I^2^: 0.00%, fixed effect). Combining transplantation to both the pelvic and ovarian sites resulted in a rate of 15% (95% CI: 0–40, I^2^: 0.00%, fixed effect). Heterotopic ovarian tissue transplantation showed a miscarriage rate of 7% (95% CI: 0–23, I^2^: 0.00%; fixed effect). The pregnancy outcomes for different sites will be summarized in Table 2.

**Table 2:** summarizes the fertility outcomes according to different transplant sites

	ovarian site	pelvic site	pelvic site + ovarian site	heterotopic transplantation
CLBR	21(6–40, I^2^: 52.81%)**	30(20–40, I^2^: 0.00%)*	23(11–36, I^2^: 0.00%)*	3(0–17, I^2^: 0.00%)*
Spontaneous LBR	16(3–35, I^2^: 0.00%)*	31(21–42, I^2^: 0.00%)*	26(12–41, I^2^: 0.00%)*	0
LBR after IVF	10(2–22, I^2^: 0.00%)*	18(7–31, I^2^: 0.00%)*	5(0–27, I^2^: 0.00%)*	3(0–17, I^2^: 0.00%)*
Women with at least one pregnancy after IVF and spontaneous pregnancy	25(13–40, I^2^: 0.00%)*	42(22–63, I^2^: 65.30%)**	25(13–38, I^2^: 0.00%)*	7(0–24, I^2^: 0.00%)*
Women with at least one spontaneous pregnancy	26(9–46, I^2^: 0.00%)*	32(14–54, I^2^: 70.92%)**	38(10–71, I^2^: 60.46%)**	0
Women with at least one pregnancy after IVF	9(0–25, I^2^: 0.00%)*	28(7–54, I^2^: 65.63%)**	15(0–40, I^2^: 0.00%)*	7(0–24, I^2^: 0.00%)*
Women with miscarriage after IVF and spontaneous pregnancy	10(2–21, I^2^: 0.00%)*	11(0–27, I^2^: 62.34%)**	8(1–18, I^2^: 0.00%)*	7(0–23, I^2^: 0.00%)*
Women with miscarriage after spontaneous pregnancy	5(0–19, I^2^: 0.00%)*	12(0–33, I^2^: 77.90%)**	3(0–12, I^2^: 0.00%)*	0
Women with miscarriage after IVF	0(0–9, I^2^: 0.00%)*	14(2–30, I^2^: 0.00%)*	15(0–40, I^2^: 0.00%)*	7(0–23, I^2^: 0.00%)*

The cumulative live birth rate (CLBR, %) and percentages of pregnancy and miscarriage were estimated for each graft site.

Percentage of events with two-sided CI estimated for all publications: heterogeneity = I^2^

*Fixed effect model

**Random effect model

### Sensitivity analysis

Six studies on the CLBR at transplanted ovarian sites were included. After the heterogeneity test, which revealed considerable heterogeneity across the literature selected for this study with I^2^ = 52.81% and P < 0.1, a sensitivity analysis was carried out to investigate the sources of heterogeneity. The sensitivity analysis of the six included studies revealed that none of them significantly affected the findings of this meta-analysis, indicating that the stability of the study was good (Supplementary Data File S3).

## Discussion

In our comprehensive meta-analysis, we estimated the CLBR, which encompassed both spontaneous pregnancies and those achieved through ART, following the transplantation of ovarian tissue to the ovarian site. Our findings indicated a CLBR of 21% (95% CI: 6–40, I^2^: 52.81%, random effect). When transplantation was performed at the pelvic site, the LBR was notably higher at 30% (95% CI: 20–40, I^2^: 0.00%, fixed effect). Similarly, when both the pelvic and ovarian sites were targeted for transplantation, the LBR remained favorable at 23% (95% CI: 11–36, I^2^: 0.00%, fixed effect). However, heterotopic ovarian tissue transplantation yielded a comparatively low LBR of 3% (95% CI: 0–17, I^2^: 0.00%, fixed effect). According to a specific study [10] that examined the number of children born following each surgical procedure, the rates of OTT to the ovary, peritoneum, and combination techniques were 30.5%, 34.8%, and 34%, respectively. These results suggest that different transplantation sites have comparable reproductive efficacies. Our findings align with this report, albeit with a slightly lower CLBR.

When oocyte or embryo cryopreservation is not possible or at the woman’s discretion, OTC according to the European Society of Human Reproduction and Embryology (ESHRE) guideline may be provided [5]. According to the American Society for Reproductive Medicine (ASRM) guidelines [33], for girls in the prepubescent stage, OTC is the only choice to maintain fertility. Before the ages of 36 and 40 years, the ESHRE and ASRM recommendations urge the use of OTC. An uncomplicated laparoscopic procedure should be used to perform OTT because it is thought to be safe and will not increase the risk of surgery [34, 35]. Children born after OTT do not have a higher chance of congenital defects [6, 36].

According to the literature, the rate of spontaneous pregnancies following OTT is higher than that for IVF following OTT [37]. Our study also revealed that spontaneous pregnancy and LBRs following orthotopic OTT were consistently higher than those achieved through IVF, regardless of the specific transplantation site. These findings suggest that IVF should not be initiated immediately when there is a possibility of spontaneous pregnancy.

Numerous factors may affect the success of transplantation, as measured by eventual pregnancy and durability of graft function. These variables include age at cryopreservation, baseline ovarian reserve, methods used to prepare and transplant tissue, history of prior cancer treatments, freeze-thaw protocols, number of grafted cortical sections, transplantation procedures, graft sites, and degree of tissue ischemia following transplantation [38]. It is imperative to emphasize two points. First, good OTT surgeries should follow basic microsurgical guidelines, which include choosing a well-vascularized transplanting site and developing a workable strategy to protect the ovarian tissue. Second, the amount of tissue chosen for transplantation should be carefully considered due to the possibility of reimplantation in the same patient in the future. When a significant amount of tissue is initially cryopreserved, such as in situations involving ovariectomy or bilateral biopsies, it is best to simply graft a portion of the cryopreserved tissue. Consideration should be given to the patient’s ovarian reserve status at the time of OTC when making this choice.

Ovarian tissue can be transplanted to heterotopic sites, such as locations outside the pelvic cavity (forearm or abdominal wall muscle) or orthotopic sites, such as the pelvic cavity (back to the medulla of the ovary or a specifically formed peritoneal pocket) [39]. Orthotopic reimplantation has proven to be the most successful surgical procedure for OTT in terms of resuming endocrine function and restoring fertility [5]. A forearm-mounted, intact, nonfrozen ovarian subcutaneous site was the first reported case of heterotopic transplantation [40]. With OTT to the forearm, follicular growth was also observed [41]; however, the dominant follicles in the two patients only grew to a maximum size of 11 mm, and no oocytes were found. Endocrine function was sporadic in both patients.

Considering the findings of this study, a notable decrease in both pregnancies and LBRs was observed following ectopic ovarian transplantation compared to in situ transplantation. These include potential surgical trauma to the ovarian tissue during transplantation, compromised blood supply to the transplanted ovaries, immune responses against the transplanted tissue, hormonal disturbances, surgical techniques, patient-specific factors, such as age and health, and the need for postoperative monitoring and additional treatments to ensure ovarian function restoration. Each case is unique, and the outcomes can vary depending on these factors. For individuals considering this procedure, it is crucial to consult experienced obstetrician-gynecologists for a personalized assessment and treatment planning, with consideration of the latest clinical research and data.

### Strengths

To our knowledge, this meta-analysis is the first to compare pregnancy outcomes at various transplant sites. A rigorous assessment of the risk of including the same population twice was performed, and sensitivity analyses were performed as necessary. Although this was a single-arm meta-analysis, the study offers comprehensive LBR knowledge and may aid professionals in counseling women. The application of a precise methodology employing PRISMA standards is another advantage. The NOS was used to evaluate the quality of the included studies, and the majority had minimal risk of bias.

### Limitations

The caliber of the studies considered in our meta-analysis determined the caliber of our analysis. Because randomized controlled trials cannot be performed in this field, only observational research is available. The experience with cryopreserved ovarian tissue autotransplantation in women with premature ovarian failure following gonadotoxic therapy is currently limited. Most of the included studies had modest sample sizes. OTT services have low return rates, ranging from 3.4–10% [42–44]. Information on patients who do not return to using their ovarian tissue is lacking. The key priority is to monitor these cohorts. Additionally, different OTC processes are employed globally, highlighting the variety of these processes. Finally, due to the popularity of fertility preservation, we were unable to include certain recent studies published after the completion of our meta-analysis. This meta-analysis highlights the need for an international register with extensive cohort follow-up. With worldwide standards requiring consistent reporting of the same characteristics, longitudinal research may be a starting point for increasing the caliber of the literature. The OTC procedure should be agreed upon and include concise steps. In general, the group of patients undergoing OTT was very diverse; some women attempted spontaneous pregnancy before IVF, while others went straight to IVF. Some women only had peritoneal grafts rather than ovarian sites, and some women had multiple transplantations, which added to the complexity of these patients.

## Conclusion

In summary, our study demonstrated that pregnancy outcomes were not significantly different after orthotopic ovarian transplantation, and that the pregnancy and LBRs after orthotopic ovarian transplantation were significantly higher than those after ectopic transplantation. Nevertheless, future large-scale and multicenter randomized controlled trials are needed to corroborate this finding die to the paucity of clinical data.

### Electronic supplementary material

Below is the link to the electronic supplementary material.

**Supplementary Material 1**: Literature search strategy

**Supplementary Material 2**: The Newcastle–Ottawa Scale (NOS)

**Supplementary Material 3**: Sensitivity Analysis

## Data Availability

The datasets used in this study can be found in the full-text articles included in systematic reviews and meta-analyses.

## List of abbreviations

OTT: ovarian tissue transplantation
CLBR: cumulative live birth rate
LBR: live birth rate
ART: assisted Reproductive Technology
CI: confidence interval
OTC: ovarian tissue cryopreservation
IVF: in vitro fertilization
OTC: ovarian tissue cryopreservation
PR: pregnancy rate
NOS: Newcastle-Ottawa Scale
O: ovarian site
P: pelvic wall
A: anterior abdominal wall
SC: subcutaneous site
HT: heterotopic transplantation
PNH: paroxysmal nocturnal hemoglobinuria
HL: Hodgkin lymphoma
NHD: non-Hodgkin disease
