# Supplementary material for: Assessing the impact of transplant site on ovarian tissue transplantation: a single-arm meta-analysis
Source: Reprod Biol Endocrinol. 2023 Dec 12;21:120. doi: 10.1186/s12958-023-01167-6 (PMC10714583; doi:10.1186/s12958-023-01167-6)
Supplement: Supplementary file 1 — Supplementary Material 1: Literature search strategy [file 12958_2023_1167_MOESM1_ESM.docx]

**Literature search strategy**：

Search: ((ovarian transplantation[Title/Abstract]) OR (ovarian tissue transplantation[Title/Abstract])) AND (((((((((Pregnancy Outcome[Title/Abstract]) OR (Pregnancy Outcomes[Title/Abstract])) OR (Outcome, Pregnancy[Title/Abstract])) OR (Outcomes, Pregnancy[Title/Abstract])) OR (reproductive outcome[Title/Abstract])) OR ((((((((((((pregnancy rate[Title/Abstract]) OR (Rates, Pregnancy[Title/Abstract])) OR (Pregnancy Rates[Title/Abstract])) OR (Rate, Pregnancy[Title/Abstract])) OR (Pregnancy Rate, Live-Birth[Title/Abstract])) OR (Live-Birth Pregnancy Rates[Title/Abstract])) OR (Pregnancy Rate, Live Birth[Title/Abstract])) OR (Pregnancy Rates, Live-Birth[Title/Abstract])) OR (Rate, Live-Birth Pregnancy[Title/Abstract])) OR (Rates, Live-Birth Pregnancy[Title/Abstract])) OR (Live-Birth Pregnancy Rate[Title/Abstract])) OR (Live Birth Pregnancy Rate[Title/Abstract]))) OR (((((((((((((((((((((((delivery rate[Title/Abstract]) OR (Perfusion Pumps, Implantable[Title/Abstract])) OR (Implantable Perfusion Pump[Title/Abstract])) OR (Implantable Perfusion Pumps[Title/Abstract])) OR (Perfusion Pump, Implantable[Title/Abstract])) OR (Pump, Implantable Perfusion[Title/Abstract])) OR (Pumps, Implantable Perfusion[Title/Abstract])) OR (Drug Delivery Systems, Implantable[Title/Abstract])) OR (Implantable Infusion Pumps[Title/Abstract])) OR (Implantable Infusion Pump[Title/Abstract])) OR (Infusion Pump, Implantable[Title/Abstract])) OR (Pump, Implantable Infusion[Title/Abstract])) OR (Pumps, Implantable Infusion[Title/Abstract])) OR (Medication Systems, Programmable Implantable[Title/Abstract])) OR (Programmable Implantable Medication Systems[Title/Abstract])) OR (Systems, Programmable Implantable Medication[Title/Abstract])) OR (Implantable Medication Systems, Programmable[Title/Abstract])) OR (Peristaltic Pumps, Implantable[Title/Abstract])) OR (Implantable Peristaltic Pump[Title/Abstract])) OR (Implantable Peristaltic Pumps[Title/Abstract])) OR (Peristaltic Pump, Implantable[Title/Abstract])) OR (Pump, Implantable Peristaltic[Title/Abstract])) OR (Pumps, Implantable Peristaltic[Title/Abstract]))) OR ((reproductive outcome[Title/Abstract]) OR (Reproductive outcomes[Title/Abstract]))) OR (((Pregnancy[Title/Abstract]) OR (Pregnancies[Title/Abstract])) OR (Gestation[Title/Abstract])))
