# Supplementary material for: Assessing the impact of transplant site on ovarian tissue transplantation: a single-arm meta-analysis
Source: Reprod Biol Endocrinol. 2023 Dec 12;21:120. doi: 10.1186/s12958-023-01167-6 (PMC10714583; doi:10.1186/s12958-023-01167-6)
Supplement: Supplementary file 2 — Supplementary Material 2: The Newcastle–Ottawa Scale (NOS) [file 12958_2023_1167_MOESM2_ESM.docx]

**The Newcastle–Ottawa Scale (NOS)**

| ID | Author, year, | country |  | Selection |  |  | Comparability |  | Outcome |  | Quality score |
| --- | --- | --- | --- | --- | --- | --- | --- | --- | --- | --- | --- |
|  |  |  | Representativeness of the exposed cohort | Selection of the non exposed cohort | Ascertainment of exposure | Demonstration that outcome of interest was not present at start of study |  | Assessment of outcome | Was follow-up long enough for outcomes to occur | Adequacy of follow up of cohorts |  |
| NO.1 | Marine Leflon, 2022 | France | ★ |  | ★ | ★ |  | ★ | ★ | ★ | 6 |
| NO.2 | R. Imbert,2014 | Belgium | ★ |  | ★ | ★ |  | ★ | ★ | ★ | 6 |
| NO.3 | C. Poirot,2019 | France | ★ |  | ★ | ★ |  | ★ | ★ | ★ | 6 |
| NO.4 | M. Vatel,2021 | France | ★ |  | ★ | ★ |  | ★ | ★ | ★ | 6 |
| NO.5 | Matthia W,2017 | Germany | ★ |  |  | ★ |  | ★ | ★ | ★ | 5 |
| NO.6 | Ina Marie Dueholm Hjorth,2020 | Denmark | ★ |  | ★ | ★ |  | ★ | ★ | ★ | 6 |
| NO.7 | Jana Liebenthron, 2019 | Germany | ★ |  | ★ | ★ |  | ★ | ★ | ★ | 6 |
| NO.8 | Ellen J. Hoekman,2019 | Netherlands | ★ |  | ★ | ★ |  | ★ | ★ | ★ | 6 |
| NO.9 | Debra Gook,2021 | Australia | ★ |  | ★ | ★ |  | ★ | ★ | ★ | 6 |
| NO.10 | Tryde Schmidt,2011 | Denmark | ★ |  | ★ | ★ |  | ★ | ★ | ★ | 6 |
| NO.11 | Tine Greve,2012 | Denmark | ★ |  | ★ | ★ |  | ★ | ★ | ★ | 6 |
| NO.12 | Genia Rozen,2021 | Australia | ★ |  | ★ | ★ |  | ★ | ★ | ★ | 6 |
