# Supplementary figures and images for: Assessing the impact of transplant site on ovarian tissue transplantation: a single-arm meta-analysis
Source: Reprod Biol Endocrinol. 2023 Dec 12;21:120. doi: 10.1186/s12958-023-01167-6 (PMC10714583; doi:10.1186/s12958-023-01167-6)

**Sensitivity Analysis**


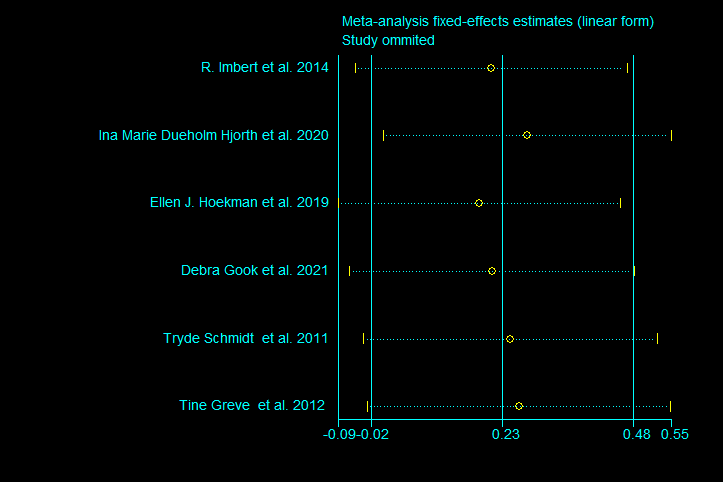

Supplement: Supplementary file 3 — Supplementary Material 3: Sensitivity Analysis [file 12958_2023_1167_MOESM3_ESM.docx]
